# Validity of a Job-Exposure Matrix for Psychosocial Job Stressors: Results from the Household Income and Labour Dynamics in Australia Survey

**DOI:** 10.1371/journal.pone.0152980

**Published:** 2016-04-06

**Authors:** A. Milner, I. Niedhammer, J.-F. Chastang, M. J. Spittal, A. D. LaMontagne

**Affiliations:** 1 Work, Health, & Wellbeing Unit, Population Health Strategic Research Centre, School of Health & Social Development, Deakin University, Melbourne, Australia; 2 Centre for Health Equity, Melbourne School of Population and Global Health, University of Melbourne, Melbourne, Australia; 3 INSERM, UMR_S 1136, Pierre Louis Institute of Epidemiology and Public Health, Department of social epidemiology, Paris, France; 4 Sorbonne Universités, UPMC Univ Paris 06, UMR_S 1136, Pierre Louis Institute of Epidemiology and Public Health, Department of social epidemiology, Paris, France; 5 Centre for Mental Health, Melbourne School of Population and Global Health, The University of Melbourne, Melbourne, Australia; University of Westminster, UNITED KINGDOM

## Abstract

**Introduction:**

A Job Exposure Matrix (JEM) for psychosocial job stressors allows assessment of these exposures at a population level. JEMs are particularly useful in situations when information on psychosocial job stressors were not collected individually and can help eliminate the biases that may be present in individual self-report accounts. This research paper describes the development of a JEM in the Australian context.

**Methods:**

The Household Income Labour Dynamics in Australia (HILDA) survey was used to construct a JEM for job control, job demands and complexity, job insecurity, and fairness of pay. Population median values of these variables for all employed people (n = 20,428) were used to define individual exposures across the period 2001 to 2012. The JEM was calculated for the Australian and New Zealand Standard Classification of Occupations (ANZSCO) at the four-digit level, which represents 358 occupations. Both continuous and binary exposures to job stressors were calculated at the 4-digit level. We assessed concordance between the JEM-assigned and individually-reported exposures using the Kappa statistic, sensitivity and specificity assessments. We conducted regression analysis using mental health as an outcome measure.

**Results:**

Kappa statistics indicate good agreement between individually-reported and JEM-assigned dichotomous measures for job demands and control, and moderate agreement for job insecurity and fairness of pay. Job control, job demands and security had the highest sensitivity, while specificity was relatively high for the four exposures. Regression analysis shows that most individually reported and JEM measures were significantly associated with mental health, and individually-reported exposures produced much stronger effects on mental health than the JEM-assigned exposures.

**Discussion:**

These JEM-based estimates of stressors exposure provide a conservative proxy for individual-level data, and can be applied to a range of health and organisational outcomes.

## Introduction

Available evidence suggests psychosocial working conditions are highly prevalent exposures in working populations around the world. In a national survey in France [1], job strain (i.e. the combination of high psychological demands and low decision latitude) affected 20% of men and 28% of women. Data from eight cohort studies in Europe indicated that the prevalence of job strain ranged from 13% in the Netherlands to 22% in Denmark [2]. Females are more likely to be exposed to psychosocial job stressors than males, at least in high-income countries such as Australia, France and Denmark [3–5]. Further, evidence suggests differences in exposure to the more commonly studied psychosocial job stressors (such as low job control) by occupation [1, 4, 6], with lower skilled occupations being disproportionately exposed to adverse working conditions than higher skilled occupations. This is the reverse for high psychological demands, as higher skilled occupations are more likely to be exposed [5].

There is growing evidence that psychosocial job stressors are associated with a range of mental and physical health outcomes. For example, several studies have shown that a combination of low social support at work, low decision latitude, high job demands, and effort-reward imbalance were together associated with an increased risk of common mental disorders [7–11]. In Denmark, high effort-reward imbalance predicted onset of severe depressive symptoms at follow-up, after adjustment for covariates and occupational grade [12]. In France, low decision latitude, high psychological demands, and low social support at work were associated with poor self-reported health for both men and women [1]. Research in Australia has demonstrated the relationship between psychosocial job quality and mental health [13], self-reported health [14], and sickness absence [15].

One of the challenges in this area of research is that many population level sources of information have limited or no information on exposure to psychosocial job stressors (PJS). This restricts study into the potential range of health outcomes connected to adverse working environments. Further, it is often difficult to compare reports of psychosocial job stressors between studies as not all data sources have the same measures of PJS. One solution to these problems is to use a Job Exposure Matrix (JEM), which can be used to assign exposures on the basis of occupational title, and is particularly useful when individual occupational information is available but there is no data on individual psychosocial job stressors.

As suggested by their name, JEMs comprise a matrix in which each intersection between rows (job titles) and columns (exposures) provides one or more estimates of average exposure, such as prevalence, frequency or duration of this exposure within each job title. Internationally, only a handful of studies have assessed the ability of JEMs to measure job stressors. One study recently conducted in Finland [16] assessed the validity of JEM estimates for job demands, job control, monotonous work and social support. The JEM showed acceptable accuracy in identification of individuals exposed to low job control and high job strain, while its performance for job demands and social support was relatively low when assessed against self-reported individual records. Another notable example of a JEM validation study was conducted in France [5, 6]. This study found that the correlation coefficients between self-reported individual scores and JEM scores were moderate for decision latitude (<0.50), and low for psychological demands (<0.32) and social support (<0.22). Results of sensitivity and specificity analyses suggested that misclassification was higher for social support, and more moderate for decision latitude and psychological demands. The results of these two studies show reliable associations between JEM measurements of job stressors and health outcomes. Further, the results mirror to those ascertained from studies using individual measurements of job stressors in relation to health outcomes [17–23]. These studies suggest that JEMs can be reliably used to estimate exposure to job stressors and highlight their usefulness in a wide range of research applications.

The aim of this study was to develop a JEM to assess psychosocial job exposures in the Australian context using the Household Income and Labour Dynamics in Australia (HILDA) study. We also assessed the degree to which JEM-assigned exposures matched individual reports of psychosocial job stressors, and compared associations of the two types of exposure estimates with a common measure of mental health.

## Method

The section below describes the data source, variables (all data on psychosocial work exposures and other variables have been collected annually), and the steps undertaken to develop the JEM. We also describe the process for assessing concordance between the JEM-assigned and individually reported exposures, as well as associations between JEM-assigned exposures and mental health.

### Data source

The Job Exposure Matrix was developed using the data from the HILDA survey. This is a longitudinal, nationally representative study of Australian households established in 2001. It collects detailed information annually from over 13,000 working age individuals within over 7,000 households [24]. The response rate in wave 1 was 66% [24]. The survey covers a range of dimensions including social, demographic, health and economic conditions using a combination of face-to-face interviews with trained interviewers and a self-completion questionnaire. Although data are collected on each member of the household, interviews are only conducted with those older than 15 years of age.

The initial wave of the survey began with a large national probability sample of Australian households occupying private dwellings [24]. Interviews were sought in later waves with all persons in sample households who had attained 15 years of age. Additional persons have been added to the sample as a result of changes in household composition with a top-up sample of 2,000 people added to the cohort in 2011 to allow better representation of the Australian population using the same methodology as the original sample (i.e., a three-stage area-based design) [25]. The response rates for new respondents who join the HILDA survey are above 70% and the (wave-to-wave) retention rate for respondents who continue in the survey is above 90% [24].

### Psychosocial job exposures used in the JEM

We identified four domains of psychosocial stress and included these in the JEM: job control, job demands and complexity, job security, and fairness of pay. These measures have been characterised psychometrically and related to mental and physical health outcomes in previous research [13, 14, 26].

Job control was measured using three items: “I have a lot of freedom to decide how I do my own work”; “I have a lot of say about what happens on my job”, and; “I have a lot of freedom to decide when I do my work”. All scale items were scaled from 1 “strongly disagree” to 7 “strongly agree”, and together had high internal consistency (*α* = 0.82). Job demands and complexity were measured using four items: “My job is more stressful than I had ever imagined”; “My job is complex and difficult”; “My job often requires me to learn new skills”, and; “I use many of my skills and abilities in my current job”. As above, items were scaled from 1 to 7 and had good internal consistency (*α* = 0.70). Job security was comprised of three items: “I have a secure future in my job”; “The company I work for will still be in business 5 years from now”, and; “I worry about the future of my job”. The first two items were scored from 1 “Strongly disagree” to 7 “strongly agree”. The last item was transformed so the direction of results went in the same direction as the others. These items had fair internal consistency (*α* = 0.64) [13]. These variables were constructed as the sum of individual items. Fairness of pay was measured with one item: “I get paid fairly for the things I do in my job” and was scored from 1 “strongly agree” to 7 “agree”. In the continuous measures, higher scores on the scales represented higher control, greater demand and complexity, greater security, and higher fairness of pay. The scales have all been used previously and have clear associations with more widely used measures of job demands and control and other employment conditions such as casual status, hours worked and shift work [14, 27].

### Other variables used in the analyses

The Mental Component Summary (MCS) of the Short Form 36 (SF-36) measure was used as the primary outcome measure in the validation analysis. The MCS score is mainly comprised of four scales: mental health, role emotional, vitality and social functioning, which are derived from 14 questions [28]. The SF-36 is a widely used self-completion measure of health status, and has been validated for use in the Australian population, and to detect within-person change over time [28]. The SF-36 in the HILDA survey has been shown to be psychometrically sound, with good internal consistency, discriminant validity and high reliability [28]. The mean score on the MCS in HILDA was 49.35, with a standard deviation of 9.45. Higher scores represent better mental health. We also included age in later regression analysis as a control variable. We stratified analysis by gender. We did not control for other variables such as occupational skill level, income or education as these would represent an overadjustment.

### Construction of the Matrix

To construct the JEM, we calculated exposure estimates at each intersection between rows (job codes) and columns (job control, job complexity and demands, job security, and fairness of pay). The matrix was developed based on exposures at the four-digit level of the Australian and New Zealand Standard Classification of Occupations (ANZSCO), which represents 358 occupation titles. The classification definitions are based on the skill level and specialisation usually necessary to perform the tasks of the specific occupation, or of most occupations in the group [29].

Data was weighted so as to provide estimates representative of the Australian working population using the cross-sectional responding person weights generated for the HILDA dataset. These weights were constructed using benchmarks from various Australian Bureau of Statistics (ABS) surveys and adjust the sample according to: sex by broad age group; state by part of state; and state by labour force status, marital status and household composition (number of adults and children) [30]. Weighting includes a ‘non-response adjustment’ from wave 2 onward, giving those people who are similar to those who did not respond in wave 1 a greater weight in subsequent waves based on a number of person and household characteristics (such as sex, age, relationship in household, location, Socio-Economic Indexes For Areas (SEIFA) index of disadvantage and dwelling type) [30].

Exposures were calculated separately by gender, recognising that exposure to psychosocial job stressors differ by gender [4]. The process that we used to calculate the JEM can be seen below:

1. Calculation of population median values
  - We inspected the overall distribution of the various stressors and based on this, decided to use the median value for each psychosocial stressor as our overall measure of the centre of the data. We inspected the overall distribution of the various stressors and based on this, decided to use the median value for each psychosocial stressor as our overall measure of the centre of the data. The median was chosen as the measure of central tendency as this is usually used in the literature on psychosocial work factors, provides balanced groups that allow to maximize statistical power, and is less influenced by outliers than the mean measure. A median score of each exposure was calculated across individuals over time in HILDA by gender.
2. Calculation of individual exposures
  - Self-reported measures of the four psychosocial work factors were used as individual scores for each exposure. Higher scores on each continuous psychosocial job exposure represented high job control, demands and complexity, security, and fairness of pay at each time point. These were calculated for individuals for each wave of data contributed. We used means as the summary measure because we were able to adjust these using the weights available in HILDA to ensure the estimates were representative of the Australian working population.
  - We then assigned each individual level score to either low or high exposure depending on the population-level medians defined above and generated this as a new variable. At the median and above, exposure was defined as low; while below the median was high exposure for control, security and fairness and the reverse for demands.
  - This then enabled us to have time-varying binary and continuously scored measures for each individual included in the data set. In the analyses assessing the validity of the JEM, only the first entry/observation for each individual was considered.
3. Calculation of JEM exposures
  - Each participant in HILDA could contribute up to 13 annual waves of data (the mean number of employed waves per person was 8.9), in up to 13 different 4-digit occupational titles. Contributed waves were sorted by 4-digit occupational title and by gender, and mean exposure values by occupational title and gender were calculated. If changes in ANZSCO codes occurred within the 13-year period for a given individual, then the observations for this individual were used to calculate JEM scores for different ANZSCO codes by gender. This resulted in a mean score for each of the four exposures by 4-digit occupational title and gender.
  - JEM binary exposures were derived from the JEM scores dichotomized at the median thresholds described above. JEM binary exposures were calculated for each ANZSCO 4 digit code and imputed to each observation according to ANZSCO code. JEM exposures were time-invariant.
  - At the last stage, we removed ANZSCO 4 digit codes with fewer than ten observations.

### Assessment of the JEM

Using an individual’s first observed entry into the study, we assessed descriptive statistics for the continuous job stressors measures. A t-test was conducted to examine potential differences in mean exposure to job stressors between males and females. We then calculated Kappa statistics assessing concordance between JEM- versus individually-assigned exposures for each psychosocial job stressor. Thresholds of the Kappa were categorized as: >0.81, very good; 0.80–0.61, good; 0.60–0.41, moderate; 0.40–0.21, low; and<0.20 bad [31]. The sensitivity and specificity of JEM binary exposures were calculated using the individual binary exposures as references (again using an individual’s first observed entry into the study).

Next, we correlated JEM binary exposure and individually reported continuous psychosocial job stressor exposures.

We then assessed the percentages of variance (R^2^) in individually reported job stressors by ANZSCO 4-digit occupational codes. The R^2^ provides the variance of the individual job stressors (scores) explained by ANZSCO occupational category.

Last, the ability to detect previously established associations between individually reported job stressors, JEM binary exposures and mental health, measured using the Mental Health Component Summary (MCS) score of the SF-36, was tested. Each exposure was studied separately. The MCS was selected because of its known associations with the HILDA psychosocial job stressors.

A linear regression analysis was used to assess the relationships between JEM binary exposures and MCS scores, controlling for age. We stratified this analysis by gender. As above, we used an individual first reported wave in HILDA to conduct this analysis. Statistical analysis was performed using STATA, version 13.1.

## Results

The sample of observations included in the creation of the JEM was restricted to those individuals in employment who had information available on psychosocial job stressors (n = 20,428). There were slightly more males than females in the sample (Table 1). Most people were employed in “professional” occupations, reflecting the general trend in the Australian employed population. The majority of people had post high-school qualifications, and about 35% had not completed high school.

**Table 1:** Characteristics of the sample, HILDA study, at their first entry wave into the study (people = 20,478).

	At entry into the study
**Age**	
Mean ± std dev	33.83 ± 14.11
**MCS**	
Mean ± std dev	48.63 ± 9.99
**SEIFA index**	
Mean ± std dev (10 point decile scale).	5.77 ± 2.93
	%
**Gender**	
Male	50.53
Female	49.47
**Occupation**	
Managers	10.39
**ANZSCO**	
Professionals	18.48
**major group**	
Technicians and Trades Workers	12.32
Community & Personal Service	11.70
Clerical and Administrative Workers	13.11
Sales Workers	14.09
Machinery Operators and Drivers	5.47
Labourers	14.43
**Education**	
Post graduate study	8.39
Bachelors degree	12.53
Diploma or certificate	28.11
Year 12	15.27
Year 11 or less	35.70

Table 2 describes the continuously scored individual exposures (in which higher scores reflected higher control, greater demand and complexity, greater security, and higher fairness of pay), the dichotomous individual exposures (low versus high exposure), and JEM assigned exposures. The population prevalence of each of these stressors is defined as the percentage of observations allocated to the “high” exposure group, based on whether individual median scores for each occupational group fell below or above the overall median across the whole HILDA population.

**Table 2:** Median, mean scores, and prevalence for individual exposures, kappa statistics, sensitivity, specificity, and correlations between individual and JEM measures of psychosocial job stressors at the four-digit ANZSCO level, measured at the time of an individuals’ first entry into the HILDA survey (people = 20,478).

	Job control	Job demands	Job security	Fairness of pay
**Male**				
**Individual exposures**				
**Minimum, Maximum**	3, 21	3, 21	3, 21	1, 7
**Median**	13	14.5	16	5
**Mean**	12.73	13.92	15.37	4.17
**(95% CI)**	(12.63, 12.83)	(13.83, 14.01)	(15.28, 15.45)	(4.07, 4.28)
**Stand. Dev.**	4.97	4.26	4.06	1.7
**Prevalence**	55.11	49.63	39.00	26.04
**ANZSCO 4 digit level**				
**Kappa**	67.21	62.63	55.82	47.17
**Sensitivity**	67.23	58.52	49.72	43.55
**Specificity**	67.16	74.62	65.56	69.56
**Corr. coeff.**	0.3375 p<0.001	0.2771 p<0.001	0.1428 p<0.001	0.1096 p<0.001
**Female**				
**Individual exposures**				
**Minimum, Maximum**	3, 21	3, 21	3, 21	1, 7
**Median**	12	13	16	5
**Mean**	11.83	13.02	15.74	4.20
**(95% CI)**	(11.72, 11.93)	(12.93, 13.11)	(15.65, 15.82)	(4.10, 4.31)
**Stand. Dev.**	4.99	4.45	3.95	1.81
**Prevalence**	62.14	40.99	34.80	27.06
**ANZSCO 4 digit level**				
**Kappa**	64.36	67.13	57.30	46.48
**Sensitivity**	60.90	63.97	51.30	56.56
**Specificity**	68.47	72.93	69.13	67.95
**Corr. coeff. (p value)**	0.3048 p<0.001	0.3615 p<0.001	0.1597 p<0.001	0.0980 p<0.001

Notes: Stand. Dev. = Standard Deviation; Corr. coeff. = Correlation Coefficient.

As can be seen in the mean exposures, males had a higher mean score of job control than females (i.e., they reported high job control). This difference was significant (12.73 versus 11.83, p<0.001). Males also reported a higher mean score on job demands and complexity than females (13.92 versus 13.02, p<0.001). Females reported slightly higher mean scores on job security than males (15.74 versus 15.37, p<0.001) and there was no difference in average exposure to fairness of pay between females and males.

Kappa statistics indicate good agreement between individual- and JEM-assigned exposures for job demands and control, and moderate agreement for job insecurity and fairness of pay. For both males and females, job control and job demands had the highest sensitivity (over 0.50), which measures the extent to which the JEM assigned exposures correctly identified people who reported being exposed (i.e. to low job control or high demands). Specificity was highest for job control and demands, i.e. with values of 0.70 or more (measuring the extent to which the JEM correctly classified those non-exposed). We also include information on the correlation between JEM assigned and individual continuous exposures. Males and females had a stronger correlation in job control (0.3375 and 0.3048) and job demands (0.2771 and 0.3615), and a low correlation in job security (0.1428 and 0.1597) and fairness of pay (0.1096 and 0.0908).

The R^2^ percentages of variance suggest that less than 17% of the variance in the individual continuous psychosocial job stressors were accounted for by ANZSCO 4 digit codes (results not shown). As above, this was calculated for individuals’ first entry into the survey. For males, the R^2^ for control was 16.8% and 14.7% for demands and complexity, and less than 1% for fairness of pay and job security. For females, the R^2^ for demands and complexity was 13.5%, and 7.9% for job control and less than 1% for fairness and pay and job security.

Table 3 shows bivariate and multivariate associations with the Mental Health Component Summary (MCS). For males, the multivariate results indicate that greater exposure to the four individually reported exposures (high demands, low control, low job security, and low fairness of pay) were significantly associated with worse mental health. JEM assigned measures of security and fairness of pay were associated with worse mental health, but were of lesser strength than the individually reported stressors. For females, the multivariate results indicate that greater exposure to low job control, low job security and low fairness of pay were associated with worse mental health, while higher psychological demands were associated with better mental health. Similar to men, the effect of JEM exposures were not as strong as individually reported exposures. The JEM-reported measure of high psychological demands was associated with worse mental health for females.

**Table 3:** Bivariate and multivariate associations of binary exposures with the Mental Health Summary (MCS) score, at the four digit level, results from a linear regression model, measured at the time of an individuals’ first entry into the HILDA survey (people = 20,478).

	Bivariate				Multivariate			
	Coef	95% CI	t value	p value	Coef	95% CI	t value	p value
**Male**								
**High psychological demands—ind. assigned**	-0.21	-0.37, -0.05	-2.60	0.009	-0.19	-0.34, -0.03	-2.32	0.020
**High psychological demands JEM assigned**	-0.26	-0.44, -0.09	-3.02	0.003	-0.12	-0.29, 0.05	-1.36	0.173
**Low job control—individual assigned**	-1.96	-2.12, -1.81	-24.58	<0.001	-1.66	-1.82, -1.50	-20.37	<0.001
**Low job control JEM assigned**	-0.29	-0.45, -0.13	-3.61	<0.001	0.07	-0.09, 0.23	0.85	0.375
**Low job security—individual assigned**	-3.91	-4.07, -3.77	-49.60	<0.001	-3.95	-4.11, -3.80	-50.28	<0.001
**Low job security JEM assigned**	-0.99	-1.15, -0.82	-11.64	<0.001	-0.79	-0.95, -0.62	-9.35	<0.001
**Low fairness of pay—individual assigned**	-2.55	-2.71, -2.39	-31.55	<0.001	-2.43	-2.60, -2.28	-30.21	<0.001
**Low fairness of pay- JEM assigned**	-1.19	-1.40, -0.98	-11.02	<0.001	-1.02	-1.23, -0.81	-9.44	0.001
**Female**								
**High psychological demands—ind. assigned**	0.01	-0.17, 0.18	0.946	0.946	0.25	0.07, 0.42	2.74	0.006
**High psychological demands JEM assigned**	-0.73	-0.90, -0.55	-8.14	<0.001	-0.23	-0.41, 0.05	-2.56	0.010
**Low job control—individual assigned**	-1.63	-1.81, -1.45	-17.88	<0.001	-1.21	-1.40, -1.03	-13.32	<0.001
**Low job control JEM assigned**	-0.26	-0.44, -0.08	-2.90	0.004	0.22	0.05, 0.40	2.51	0.012
**Low job security—individual assigned**	-3.61	-3.79, -3.44	-40.31	<0.001	-3.54	-3.72, -3.37	-40.10	<0.001
**Low job security JEM assigned**	-1.31	-1.54, -1.09	-11.45	<0.001	-0.91	-1.13, -0.69	-7.98	0.001
**Low fairness of pay—individual assigned**	-2.60	-2.78, -2.42	-28.56	<0.001	-2.53	-2.70, -2.35	-28.11	<0.001
**Low fairness of pay- JEM assigned**	-0.68	-0.95, -0.42	-5.11	<0.001	-0.74	-1.00, -0.48	-5.62	<0.001

Note: multivariate adjusts for age, each psychosocial work exposure studied separately, high scores on the MCS = better mental health. Coef = Coefficient; 95% CI = Upper and lower confidence intervals at 95% significance; p value = significance at 95%. Exposures were used as binary variables.

## Discussion

This study has described the development of a Job Exposure Matrix (JEM) for the measurement of psychosocial job stressors in the Australian context using the HILDA study. We developed this across 358 occupational job titles for four exposures: job control, job demands and complexity, fairness of pay, and job security. We conducted internal validation within the HILDA data set and found that the JEM-based exposures had moderate to acceptable agreement with individually reported exposures. However, the job demands and complexity and job control measure performed better in the validation than did job security and fairness of pay. We would suggest that this indicates that this is because the former are more likely to reliably pattern by occupation code than the latter, thus might be more amenable to measure in a JEM.

A number of studies have recognised that psychosocial job stressor JEMs may be particularly applicable for mental health outcomes such as anxiety, depression and dementia [6, 32, 33]. In Denmark, a JEM has been applied to assess psychosocial working conditions in a population-based nested case-control study of 14,166 psychiatric patients compared with 58,060 controls. Results of this study suggest that psychosocial job stressors such as low job control were associated with an increased risk of anxiety disorders in men, while high emotional demands in a job were associated with an elevated risk of depression among women. In both sexes, high demands were associated with a decreased risk of anxiety disorders [33]. In France, a JEM measuring psychosocial job stressors found small but significant and systematic associations between job strain (the combination of low job control and high demands) and depressive symptoms among men across the working population [6]. A German study [32] used a JEM to study the possible influence of work factors in relation to dementia. The use of a JEM eliminates substantial methodological challenges in this area of work, as dementia patients are not usually able to report previous exposures accurately and interviews with next-of-kin are known to be subject to recall bias. Results suggest that there were dementia-protective associations for high challenge at work, high control possibilities at work, and high social demands at work. High risks for error at work revealed a significant positive association with the diagnosis of dementia. The findings from these JEM based studies and this one have been consistent with job stress theory as well as with results of other studies [34], supporting the validity of this exposure assessment method. Psychosocial job stressors measured using imputation methods such as JEMs have also been used to study physical health outcomes, such as cardiovascular disease [35] and all-cause mortality [17]. This highlights the important range of outcomes that JEM-based measures can be used to study. Understanding the role of job stressors (as measured through a JEM) on a wider range of health outcomes is important in formulating targeted and specific prevention plans.

The limitations of our study include the fact that it only provides information on a small number of exposures. For example, we do not have any measure of supervisor or collegial workplace social support. This is because of restrictions in the amount of data and variables available in HILDA. In addition, we have generated a JEM with averaged exposures over a 12-year observation period; with an acknowledged limitation that some psychosocial stressor exposures may have varied by time period. Another issue is the potential for exposure misclassification within occupational titles (as all persons in a given occupation are assigned the same exposure value, while acknowledging that when measured at the individual level exposures can vary within occupation). At the same time, the use of a JEM helps in the case of missing exposure data. Further, JEM assessments provide an objective measure of exposure information by summing observations across all people having the same occupation, overcoming the potential for individual reporting bias and spurious associations with self-reported outcomes due to common method variance, or dependent misclassification [36]. Regarding the data source, we would highlight that HILDA is slightly biased towards more educated, and highly skilled workers. However, we adjusted for this potential distortion by using the weights available in the HILDA dataset, making HILDA a large nationally representative survey of the Australian population, pertinent to evaluate psychosocial work exposures at the national level. It is also a problem that we could not validate the JEM against an independent sample, and would suggest this as a future area of research.

Our results suggest that a JEM to measure psychosocial job exposures produces acceptable ratings when compared to individual reported exposures. However, our study in relation to MCS showed that JEM-generated exposures yield associations that are substantially biased toward the null. This introduces the possibility of misclassification, but as this non-differential (i.e., all occupations are equally likely to be affected), these associations maybe more likely to be bias towards the null. However, we would note that results for JEM- generated fairness of pay was significant, despite having low agreement with individual measures and a lower prevalence than the other stressors. A plausible explanation for this is greater likelihood of misclassification due to its lower prevalence, which may be biasing results away from the null. It is also important to note that the JEM performed better with job demands and control, than it did with fairness of pay and job security. As we note above, this is likely to suggest that demands and control pattern by occupational code more so than fairness of pay or job security. Thus, JEM measures of these latter two stressors are not as reliable as for the former. We would also acknowledge that the overall internal consistency of the job security scale was lower than compared to demands and control, which represents a further limitation and cautions against further research with this scale.

We would recommend the development of the JEM able to measure a wider number of psychosocial job exposures than measured in our study. This could be done in two ways. One, by developing a custom-built JEM for the Australian context, which would require (preferably) longitudinal data on various psychosocial exposures ascertained from a representative sample of the working population. Careful attention should be given to the representative nature of the sample, in order to provide estimates of job stressor exposures across all working people in the Australian population. The development of a new JEM would also require further validation. A second option would be to adapt an existing JEM to the Australian context. For example, using one of the overseas JEMs with information on psychosocial job exposures that have been used in mental health studies overseas [6, 32, 33]. The benefit of this approach is that results would be comparable with existing studies overseas. However, there would still need to be work in validating these exposures in the Australian context in order to ensure that these are relevant to Australian workplaces. We would also suggest that researchers do future research on the ability of JEMs to measure different types of job stressors. As above, we found that demands and control were more reliably measured than fairness of pay and job security.

## Conclusion

The use of a JEM is useful because it widens the range of outcomes in which psychosocial job exposures can be studied, thus this is valuable for future health research. In particular, JEMs can be used with existing data sources that have valuable outcome data as well as information on occupation in the absence of job stressor exposure data. Currently there are only a handful of JEMs internationally with available information on psychosocial job exposures, thus this is a novel and important contribution to research. As long as there is available information on occupation, a JEM could be used to study a diverse range of physical and mental health outcomes. The next steps in our work is to validate the JEM using other nationally representative data sources, and, following this to implement in further studies on mental health. Long term, research using JEM-based exposures will enable us and others to inform public health policy about the relationship between job stressors and health outcomes, leading to the development of new population-based intervention strategies.
